# Health-Related Quality of Life Assessed in Children with Chronic Rhinitis and Sinusitis

**DOI:** 10.3390/children8121133

**Published:** 2021-12-04

**Authors:** Lechosław Paweł Chmielik, Grażyna Mielnik-Niedzielska, Anna Kasprzyk, Tomasz Stankiewicz, Artur Niedzielski

**Affiliations:** 1 Department of Pediatric Otolaryngology, Centre of Postgraduate Medical Education, 01-809 Warsaw, Poland; anna.kasprzyk@g.pl (A.K.); arturniedzielski@wp.pl (A.N.); 2 Department of Pediatric ENT, The Hospital’s Pediatric in Dziekanow Lesny, 05-092 Dziekanów Leśny, Poland; 3 Department of Pediatric Otolaryngology, Medical Uniwersytety of Lublin, 20-093 Lublin, Poland; grazyna.niedzielska@wp.pl; 4 Independent Otoneurological Laboratory, Medical Uniwersytety of Lublin, 20-093 Lublin, Poland; tstankiewicz@o2.pl

**Keywords:** health quality of life, CHQ-PF-50, chronic rhinosinusitis, children, chronic sinusitis

## Abstract

Introduction: Quality of life (QoL) can be simply defined as an area of human life that directly affects people which they consider to be important. This can be defined in greater detail as ‘an individual perception of an individual’s life position within a cultural context, value system and in relation to their tasks, expectations and standards determined by environmental conditions’. The health-related quality of life (HRQoL) more specifically focuses on how the QoL affects health (including both medical and non-medical issues). Limitations in well-being will, by association, also occur in those children suffering with sinus diseases. Study aim: To compare the quality of life in children–adolescents suffering from some of the most commonly occurring childhood diseases of chronic rhinitis and sinusitis on a group of healthy children–adolescents. Test materials and methods: Subjects were children–adolescents with at least one of the aforementioned conditions afflicting the upper respiratory tract. Admission criteria were: ages 5 to 18 years in the presence of a chronic disease such as chronic rhinitis and paranasal sinusitis. The Child Health Questionnaire-Parent Form 50 CHQ-PF-50 (CHQ-PF50) was used, which is a general-purpose research instrument based on psychometric testing designed for assessing physical and mental well-being in children–adolescents aged 5 to 18 years. Results: Wellbeing significantly deteriorated in sick children within the following areas: current health status of the child (STAND), physical fitness (PF), social functioning resulting from behaviour or emotional state (REB), the impact of physical health on limitations in social functioning (RP), pain and discomfort (BP), behaviour (BE), mental health (MH), self-esteem (SE), general health perception (GH), influence of the child’s health condition on parental emotions (PE), limitations on parental leisure time due to the child’s health (PT) and restrictions on joint family activities (FA). Conclusions: The greatest impairment to well-being in children with chronic rhinitis and paranasal sinusitis was on the impact of the child’s health status on parents’ emotions, pain and discomfort and general perception of health. This study confirms that parents of healthy children attach great importance to their health and health-related quality of life.

## 1. Introduction

The quality-of-life issue only really started to be dealt with in the second half of the 20th century. Initially, it was used as a criterion to measure the level of human progress in the United States of America and Europe, where only objective parameters had been assessed, such as material goods. Hitherto, subjective ones were introduced, (i.e., non-material parameters), such as health, freedom and happiness. Over time, more and more attention has been paid to these subjective parameters in quality-of-life assessments [1,2,3,4]. Moreover, many attempts have been made to define the quality of life more strictly and uniformly; nonetheless, there is as yet no generally accepted definition [3,5,6]. According to the WHO definition from the 1940s, the term ‘health’ not only means the absence of disease, but also the coexistence of physical, mental and social well-being [7]. In order to focus more on health and medical issues, the HRQoL concept has since been developed, which deals with illness and the effect of treatment in terms of how patients/individuals view their health together with the other related QoL aspects of their lives that may also affect their health, such as work, friendships, family and other circumstances. Factors such as freedom, earnings or the natural environment are excluded [2,8,9]. The HRQL is a holistic approach embracing human health as a whole, which appeared in the 1970s [10]. Any treatment should thus make a patient’s active life as close as possible to that of a healthy person [8,9].

### Definitions of Quality of Life

Simply put, quality of life encompasses those areas of human life that directly concern a given person and are important [7]. In 1990, *Schipper* introduced the concept of a health-related quality of life, which was defined as ‘the functional effects of an illness and its consequent therapy upon a patient, as perceived by the patient’ [1,6]. From this perspective, the health-related quality of life includes the following four areas: physical condition and mobility, mental state, social status and social acceptance. Chronic rhinitis and paranasal sinusitis are inflammatory processes of the mucosa and are both regarded as being a heterogeneous disease in children. Their prevalence is currently estimated at 4% in children [10]. Both passive and active smoking are associated with chronic rhinitis and chronic rhinitis in children [11]; however, so far, allergic rhinitis has not been confirmed to predispose patients to chronic rhinitis (CR) [12]. Evidence suggests that the adenoid constitutes a reservoir for pathogenic bacteria rather than an obstruction of the upper respiratory tract [13,14]. However, the relationship between gastro-oesophageal reflux disease with CR in children is still under discussion [12,15]. There is much evidence to show a familial predisposition to CR in children [16]; nevertheless, studies on monozygotic twins have not demonstrated any similarities in the incidence of polyps, suggesting that this could be influenced by both environmental and genetic factors. This, however, causes difficulties in disease classification, therapy and diagnosis. Normal functioning of the paranasal sinuses may also be affected by pathologies of the upper respiratory tract such as adenoid hypertrophy or curvature of the nasal septum. Furthermore, during therapy, any strategy adopted will be affected by the coexistence of other pathological factors. The heterogeneous nature of the course of chronic sinusitis poses difficulties that are reflected in the lack of there being any uniform classification of sinusitis for children. A modified classification by Meltzer [17], however, appears to be the most useful way during child development, as follows:

Acute inflammation: the inflammatory process lasts up to 4 weeks, does not leave permanent traces in the mucosa and is treated conservatively. Recurrent acute sinusitis: episodes of acute sinusitis of at least five times a year. Chronic inflammation: an infectious condition of the nasal mucosa and sinuses typical in children which occurs during the cold season and resolves spontaneously in the summer and after the child’s immune system reaches maturity. Chronic inflammation: an inflammatory process lasting more than 12 weeks, which, after conservative treatment, leaves permanent lesions in the mucosa.

The EPOS 2020 [18] classification is used clinically to diagnose chronic sinusitis, with or without polyps, when at least two symptoms are present (i.e., the so-called main symptoms), from which nasal obstruction, discharge in the nasal cavities or at the back of the throat, facial pain and a feeling of fullness and an impairment or loss of smell occur. These symptoms must last more than 12 weeks. Treatment of chronic rhinitis and paranasal sinusitis in children should be multifaceted (wide ranging) and must include antibacterial, immune-modulating and local-symptomatic treatment. Nevertheless, it must a priori remove the cause of the disease, i.e., any factor impeding the patency of the nose or paranasal sinuses. This usually requires surgery.

Studies have been published on HRQL related to chronic sinusitis, wherein the quality of life has been described to be limited to areas such as general health, pain, discomfort, the effect of the child’s health status on parents’ emotions, physical fitness, limitations in social functioning, limitations on parents’ leisure time and mental health [9,17,18,19]. However, it was found that this disease condition does not limit self-esteem and does not change behaviour [20,21]. Patients with chronic rhinitis and paranasal sinusitis have a long-term impaired nasal patency, whilst ENT examination shows discharge in the nasal cavities or at the back of the throat. A child also feels pain, a feeling of fullness in the face, and parents report a loss or impairment of smell [22].

Any given study aim determines how an appropriate questionnaire is chosen. General-purpose questionnaires are used to study large populations with a variety of pathologies. It is thereby possible to compare study outcomes with each other, regardless of whether the examined subjects are healthy or suffering from any medical conditions. Different-sized groups can also be compared. As research tools, however, general-purpose questionnaires are not useful for assessing discrete changes in an individual. Specific HRQL research questionnaires have been so designed for specific areas under study. These are more sensitive in detecting deviations occurring over time and are therefore used for investigating the efficacy of treatment or the progression of lesions. They are, however, unsuited for studying patients suffering from several diseases at once (comorbidities).

This study aims to assess the quality of life in those children with chronic rhinitis and paranasal sinusitis compared to a group of healthy children.

## 2. Study Materials and Methods

The study’s test group included child subjects suffering from one of the most common chronic diseases of the upper respiratory tract: chronic rhinitis and paranasal sinusitis. The admission criteria were ages 5 to 18 years and the presence of a chronic disease such as chronic rhinitis and paranasal sinusitis. All children diagnosed with CR were based on EPOS criteria. Exclusion criteria were ages under 5 and over 18 years and having acute childhood diseases or an uncompleted questionnaire. The control group were from children–adolescents attending nursery school, primary school, middle school and high school in Warsaw and its surrounding regions. School institutions and child–adolescent subjects were randomly selected from a list of schools with assigned numbers using a random number generator program. Children–adolescents, assigned by number codes, were then chosen randomly using said program. The criteria for admission to the control group were, as before, ages from 5 to 18 years, whilst exclusion criteria were ages under 5 and over 18 years, having acute childhood diseases and chronic childhood diseases or an incompletely filled-in questionnaire. All questionnaires were completed by the children–adolescent’s parents regardless of the age group of the respondent.

The study was performed using the Child Health Questionnaire—Parent Form 50 CHQ-PF-50 (CHQ-PF50), which is a general-purpose research tool based on psychometric testing, in order to assess physical and mental well-being amongst children–adolescents aged 5 to 18 years. This has been used to measure health-related quality of life in both healthy and sick children since 1994, when it had then been introduced by JM Landgraff and JE Ware [19]. The questionnaire was constructed based on the assumption that the health status is assessed on physical and mental well-being (including areas related to emotions, behaviour and social contacts). The questionnaire consisted of 13 categories of 50 questions answered by parents/legal guardians. The duration of assessment depended on the group of questions. Areas of general perception regarding health and family cohesion did not have any specific time frame. The current health status was compared to that of one year ago. The last four weeks were, however, only investigated in the remaining question groups. Each answer was expressed as an appropriate numerical value. Results were calculated by the following algorithm: the sum of obtained values divided by the number of questions answered, from which the lowest possible value is then subtracted. This obtained value was next divided by the range of possible outcomes. The final scores ranged between 0 and 100 [23]; the higher the score, the better the life functioning and well-being.

### 2.1. Statistical Analysis

This was performed using the STATISTICA package. Significance was taken as being *p* = 0.05 whilst choosing two-tailed tests were performed at the researcher’s discretion. Table values of *p* of less than 0.05 are denoted in red.

### 2.2. Variables

These were all described in the questionnaire and were divided into two main groups: discrete and continuous. Discrete variables were further divided into those variables with two-point distributions and ones with n-point distributions. For each discrete variable, the counts and structure indices were calculated. Basic summary statistics were calculated for each continuous variable: count, arithmetic mean, standard deviation, minimum value, maximum value, skewness and kurtosis indices, as well as positional statistics: median, Q25, Q75. Most of the continuous variables deviated from the normal distribution, which thereby required using non-parametric methods for performing the statistics; however, parametric tests were also sometimes employed as part of data mining.

#### 2.2.1. Continuous Variables, Median Analysis, Analysis of Averages, Correlation

The non-parametric tests used were the Mann–Whitney test (with corrections for associated ranks), the Kruskall–Wallis test to compare mean ranks and the median test. Two parametric tests were used, as appropriate: the Student’s *t*-test or one-way analysis of variance. The first was used when the grouping variable had a two-point distribution. Equality of variance was tested by the F-test. If the grouping variable had a different distribution, analysis of variance (ANOVA) was used. Equality of variance was checked by the Brown–Forsythe test. The RIR–Tukey test was used for multiple comparisons. Spearman’s rank correlation coefficient and Tukey’s correlation coefficient were used to calculate the correlation.

#### 2.2.2. Discrete Variables

The independence analysis used the chi-square test of independence. A two-tailed exact test was used in the case of 4-field tables whenever numbers were smaller than expected. Appropriate groupings were used for tables with more fields. For the purposes of interpretation, Wanke’s surplus values were also calculated in the contingency tables.

## 3. Results

The control group consisted of 50 girls and 52 boys with an average age of 10.58 years; the youngest being 5 years old and the eldest 18 years old (Table 1).

Mean values ranged from 3.78 to 97.11 for continuous variables in the control group, whereas the standard deviation was between 0.86 and 14.21 and the median ranged from 4.40 to 100.00. There were 150 CHQ-PF-50 questionnaires distributed to parents of the test group of children (i.e., those with chronic rhinitis and paranasal sinusitis), out of which 102 (68%) questionnaires met the admission criteria. This group was composed of 51 girls and the same number of boys with a mean age of 10.84 years; the youngest were 5 years old the oldest were 18 years old (Table 1).

The mean values ranged from 3.34 to 86.06 for continuous variables in the group of children with chronic rhinitis and paranasal sinusitis, whilst standard deviations ranged between 0.84 and 28.46 and medians ranged from 3.40 to 100.00 (Table 2).

There were no statistically significant differences when comparing healthy children–adolescents and those with chronic rhinitis to paranasal sinusitis using the median test within the area of well-being of (FC) family cohesion. However, significant differences in deteriorating well-being were found in sick children in the following areas: assessment of the current health status of the child (STAND), physical fitness (PF), social functioning resulting from behaviour or emotional state (REB), the impact of physical health on limitations in social functioning (RP), pain and discomfort (BP), behaviour (BE), mental health (MH), self-esteem (SE), general health perception (GH), influence of the child’s health condition on parental emotions (PE), limitations on parental leisure time due to the child’s health (PT) and restrictions on joint family activities (FA) (Table 3).

## 4. Discussion

The presented study showed mean values for individual quality of life parameters to range from 53.18 to 86.06 in a group of children–adolescents suffering from chronic rhinitis and paranasal sinusitis. All children diagnosed with CR were based on EPOS criteria. The greatest reduction (53.18) was found to be in the perception of general health (GH) 53.18 whilst the impact of the child’s health on parental emotions (PE) was equally low at 56.86 and pain and discomfort (BP) was at 57.45, demonstrating the significant impact of the above-mentioned elements on the health-related quality of life. In contrast, social functioning resulting from behaviour or emotional state (REB) was rated the best at 86.06 whilst physical fitness (PF) also scored highly at 83.88.

Parental replies received from subjects diagnosed with chronic rhinitis and paranasal sinusitis showed a statistically significant deterioration in wellbeing when compared to healthy children from the control group in the following areas: current general health status (STAND), physical fitness (PF), social functioning resulting from behaviour or emotional state (REB), the impact of physical health on limitations in social functioning (RP), the occurrence of pain and discomfort (BP), behaviour (BE), mental health (MH), self-es-teem (SE), general health perception (GH), the impact of children’s health on parental emotions (PE), limitations in parental leisure time due to children’s health (PT) and limitations in joint family activities (FA). There were, however, no significant differences in family cohesion (FC). To the best of our knowledge, there are no studies in the Polish literature dealing with the quality of life in children with chronic rhinitis and paranasal sinusitis, whilst we found only one reference in the world literature that assessed HRQL in a small group of children with this diagnosis in a study by Cunningham et al. This used a general-purpose questionnaire on 21 children in the USA treated by endoscopic surgery of the paranasal sinuses for CRS. The questionnaires were CHQ PF-50 and CHQ CF-87, taking into account the self-esteem of the patients and the assessment made by the parents of their children.

The study demonstrated significant limitations in the quality-of-life profile of children with CP compared to children with other chronic diseases such as asthma, epilepsy, rheumatoid arthritis and mental disorders. HRQL was particularly low in the following areas: the impact of physical health on limitations in social functioning (RP) and pain and discomfort (BP). They also found that children with CP gave more favourable self-assessments of their individual quality of life indicators than those of their parents. Nevertheless, decreases were observed in both children’s and parents’ responses concerning the perception of general health (GH), pain and discomfort (BP), physical fitness (PF), limitations in social functioning (REB/RP), limitations in parental leisure time due to children’s health (PT) and mental health (MH). However, there were no constraints observed in self-esteem (SE) or behaviour (BE). Such outcomes are mostly consistent with those found in the presented study, differences being in an unchanged family cohesion (FC) compared to controls in the presented study (as opposed to a deterioration in the quoted work) as well as in a decreased self-esteem (SE) and behaviour (BE) in our study.

It should, however, be remembered that assessing the quality of life of both a healthy child and a sick child, according to the individual health-related elements, also depends on those socio-economic and cultural conditions prevailing in individual countries. Another factor is that the sizes of the subject groups were different in each study. It is also impossible to compare changes in the quality of life in health or ill children when using specific questionnaires intended solely for assessing any given disease. Current studies in the literature have assessed the quality of life using specific testing for laryngological diseases and have shown a worse quality of life in children with CP in terms of their life activity, emotional disorders, allergy symptoms, nasal blockage and symptoms of sinus infections [18,24]. Such outcomes essentially agree with the presented study, although drawing conclusions from any comparison is precluded because of the significant differences in the methodologies employed.

The quality of life for adults is assessed in the EPOS2020 classification. An overall assessment of symptom severity can obviously be high depending on the population studied. Those CRS patients identified from the general population had achieved mean scores of 8.2 and 7.8 for, respectively, CRSwNP and CRSsNP (on the VAS scale ranging from 0 to 10), when asked how bothersome/irksome their symptoms were in general. Moderate-to-severe average symptom scores were reported by hospital patients awaiting surgery; with a mean SNOT-22 score of 42.0 [18] compared with controls at 9.3. A median score of 7.0 has been proposed as a threshold for normal scores [19], where patients with CRSsNP have higher pre-operative baseline scores 44.2 compared to CRSwNP 41.0.

CRS has been shown to affect patients’ health-related quality of life. There are significant differences in all domains of the SF-36 when compared to healthy controls [25]. A landmark paper by *Gliklich* and *Metson* demonstrated, for first time, the impact of CRS on global quality of life, where it was stated that CRS has a greater impact on social functioning than angina or chronic heart failure [19]. They have also recently shown that health utility values, as measured by the EQ-5D, were lower than in the general population and indeed comparable to other chronic diseases such as asthma [25]. The severity of symptoms is influenced by gender, with greater severities seen in women that impact their quality of life, as measured by disease-specific tools or by global measures such as SF-36 or Eq-5D [20,25]. Coexisting depressive disease is associated with a poorer quality of life specific to CRS [19]. The severity of symptoms may depend in part on the severity of the disease but is further affected by the patient’s self-characteristics (e.g., gender, ethnicity, religious and cultural beliefs) as well as comorbidities and external characteristics such as socioeconomic factors and support systems. This likely explains the mismatch commonly found between objective and patient-assessed disease severity scales, such as those assessing radiological severity and symptoms [21,26,27,28].

## 5. Conclusions

Children suffering from chronic rhinitis and paranasal sinusitis demonstrate that the greatest limitations/constraints to their well-being occurs in areas where the child’s health status affects their parents’ emotions, and negative feelings of pain discomfort and general perception of health. The health-related quality-of-life assessment demonstrates that healthy children parents attach great importance to the health of their children.

## Figures and Tables

**Table 1:** Summary statistics—continuous variables for the control group.

Control	N	Mean	Std. Dev.	Min.	Q25	Median	Q75	Max.	Skewness	Kurtosis
STAND (Current health status)	102	3.78	0.86	1.00	3.40	4.40	4.40	5.00	−0.88	0.11
PF (physical functioning,)	102	97.11	5.17	77.78	94.44	100.00	100.00	100.00	−2.01	3.69
REB (Behavioural/emotional state)	102	96.51	7.49	66.67	100.00	100.00	100.00	100.00	−2.29	4.85
RP (Social functioning)	102	96.24	9.92	50.00	100.00	100.00	100.00	100.00	−2.75	7.09
BP (Pain and discomfort)	102	85.39	16.75	10.00	70.00	90.00	100.00	100.00	−1.37	2.94
BE (Behaviour)	102	79.19	11.15	55.00	71.67	80.83	89.17	100.00	−0.41	−0.60
MH (Mental Health)	102	79.80	13.62	30.00	70.00	80.00	90.00	100.00	−0.87	0.52
SE (Self esteem)	102	80.19	14.07	37.50	70.83	83.33	91.67	100.00	−0.91	0.59
GH (General health perception)	102	75.41	13.12	29.17	68.33	76.67	85.00	100.00	−1.08	1.52
PE (Parental emotions)	102	77.21	14.21	41.67	66.67	75.00	91.67	100.00	−0.30	−0.47
PT (Child’s health)	102	90.41	11.60	66.67	88.89	88.89	100.00	100.00	−0.97	−0.30
FA (Joint family activities)	102	85.29	12.90	50.00	75.00	89.59	95.83	100.00	−0.78	−0.19
FC (Family cohesion)	102	66.57	18.66	0.00	60.00	60.00	85.00	100.00	−0.76	0.77
AGE	102	10.58	3.55	5.00	8.00	9.00	13.00	18.00	0.80	−0.41

* The following is a list of abbreviations used in the table below: PF—physical functioning, RP—role/social–physical, GH—general health perceptions, BP—bodily pain/discomfort, PT—parental impact-time, PE—parental impact-emotional, REB—role/social emotional–behavioral, SE—self esteem, MH—mental health, BE—general behavior, FA—family limitations in activities, FC—family cohesion, STAND—assessment of the general condition of the child.

**Table 2:** Summary statistics—continuous variables for the test group (chronic rhinitis and paranasal sinusitis).

Control	N	Mean	Std. Dev.	Min.	Q25	Median	Q75	Max.	Skewness	Kurtosis
STAND (Current health status)	102	3.34	0.84	1.00	3.40	3.40	3.40	5.00	−0.68	0.96
PF (physical functioning)	102	83.88	18.02	27.78	72.22	88.89	100.00	100.00	−1.25	0.99
REB (Behavioural/emotional state)	102	86.06	23.38	0.00	77.78	100.00	100.00	100.00	−1.92	3.28
RP (Social functioning)	102	76.63	28.46	0.00	66.67	83.33	100.00	100.00	−1.28	0.82
BP (Pain and discomfort)	102	57.45	25.51	0.00	40.00	50.00	70.00	100.00	0.14	−0.57
BE (Behaviour)	102	70.57	15.88	25.83	58.33	72.50	80.83	100.00	−0.18	−0.29
MH (Mental Health)	102	66.37	17.45	20.00	55.00	65.00	80.00	100.00	−0.21	−0.24
SE (Self esteem)	102	73.41	16.65	12.50	66.67	75.00	87.50	100.00	−0.76	0.88
GH (General health perception)	102	53.18	16.56	5.00	43.33	51.67	64.17	91.67	−0.11	−0.04
PE (Parental emotions)	102	56.86	22.08	0.00	41.67	58.33	75.00	100.00	−0.01	−0.39
PT (Child’s health)	102	73.75	23.91	11.11	55.56	77.78	100.00	100.00	−0.61	−0.68
FA (Joint family activities)	102	73.37	19.96	16.67	58.33	75.00	87.50	100.00	−0.72	0.01
FC (Family cohesion)	102	63.58	22.84	0.00	60.00	60.00	85.00	100.00	−0.58	0.30
AGE	102	10.84	3.28	5.00	8.00	11.00	14.00	18.00	−0.03	−1.13

**Table 3:** A comparison of children suffering from chronic rhinitis and paranasal sinusitis (test group) with controls.

	Control Means	Test Means	Control Medians	Test Medians	*p* Medians
STAND (Current health status)	3.78	3.34	4.40	3.40	0.0000
PF (physical functioning,)	97.11	83.88	100.00	88.89	0.0000
REB (Behavioural/emotional state)	96.51	86.06	100.00	100.00	0.0093
RP (Social functioning)	96.24	76.63	100.00	83.33	0.0000
BP (Pain and discomfort)	85.39	57.45	90.00	50.00	0.0000
BE (Behaviour)	79.19	70.57	80.83	72.50	0.0000
MH (Mental Health)	79.80	66.37	80.00	65.00	0.0000
SE (Self esteem)	80.19	73.41	83.33	75.00	0.0007
GH (General health perception)	75.41	53.18	76.67	51.67	0.0000
PE (Parental emotions)	77.21	56.86	75.00	58.33	0.0000
PT (Child’s health)	90.41	73.75	88.89	77.78	0.0015
FA (Joint family activities)	85.29	73.37	89.59	75.00	0.0004
FC (Family cohesion)	66.57	63.58	60.00	60.00	0.4678

* Table values of *p* of less than 0.05 are denoted in red.

## Data Availability

The data supporting reported results can be found at correspondent author.

## Abbreviation

CHQ-PF-50	Child Health Questionnaire-Parent Form 50
CRS	Chronic rhino sinusitis
PF	Physical Functioning
RP	Role/Social-Physical
GH	General Health Perceptions
BP	Bodily Pain/Discomfort
PT	Parental Impact-Time
PE	Parental Impact-Emotional
REB	Role/Social Emotional-Behavioral
SE	Self Esteem
MH	Mental Health
BE	General Behavior
FA	Family Limitations in Activities
FC	Family Cohesion
STAND	Assessment of the general condition of the child
